# Multiple Myeloma with Primary Manifestation in the Mandible

**DOI:** 10.7759/cureus.2265

**Published:** 2018-03-03

**Authors:** Ibrahim K Ali, Amit R Parate, Vikrant O Kasat, Amaresh Dora

**Affiliations:** 1 Oral Medicine and Radiology, Nair Hospital Dental College; 2 Oral Medicine and Radiology, Govt. Dental College, Aurangabad

**Keywords:** multiple myeloma, malignancy, metastasis

## Abstract

Multiple myeloma (MM) is a malignant neoplasm of plasma cell origin. It usually has a multicentric origin within the bone. It contributes to about 1% of all malignancies and 15% of all hematologic malignancies. There is a monoclonal proliferation of abnormal plasma cells in this disease that results from a single malignant precursor that has undergone an uncontrolled mitotic division. Later, these cells produce one type of immunoglobulin light chain, either kappa or lambda. We present a case of a 46‑year‑old male patient who presented with a swelling of the mandible. The punched-out radiolucencies in the skull radiograph and the immunohistochemistry confirmed the case as MM.

## Introduction

Multiple myeloma (MM) is a plasma cell neoplasm characterized by the multicentric proliferation of plasma cells in the bone marrow. Multiple myeloma is a relatively rare malignant hematological disease, with an unknown etiology [1]. It occurs commonly between 50 to 80 years of age and occurs twice as often in men as in women. The most frequent clinical signs and symptoms of multiple myeloma consist of anemia, bone pain, fatigue, and infections, and it is characterized by multiple punched-out radiolucent lesions [2]. Maxillofacial manifestations of multiple myeloma are seldom present as an initial sign but may present as a primary manifestation in the advanced stages of the disease [2-3]. The maxillofacial lesions are more common in the posterior region of the mandible, manifesting as odontalgia, paresthesia, dental mobility, gingival hemorrhage, ulcerations [4]. The clinical features are the consequences of the prolifera­tion and expansion of neoplastic plasma cells in the bone marrow along with the excessive production of immunoglobulins, which often have abnormal physicochemical properties. The primary symptom is related to the bone destruction caused by tumor cells. This disease accounts for about 1% of all malignancy and 10% of hematologic malignancy [4-5]. We describe a case of multiple myeloma involving the mandible in a 46-year-old man who experienced swelling in the right mandibular alveolar region along with a metastatic lesion involving the acromioclavicular joint.

## Case presentation

**Figure 1:**
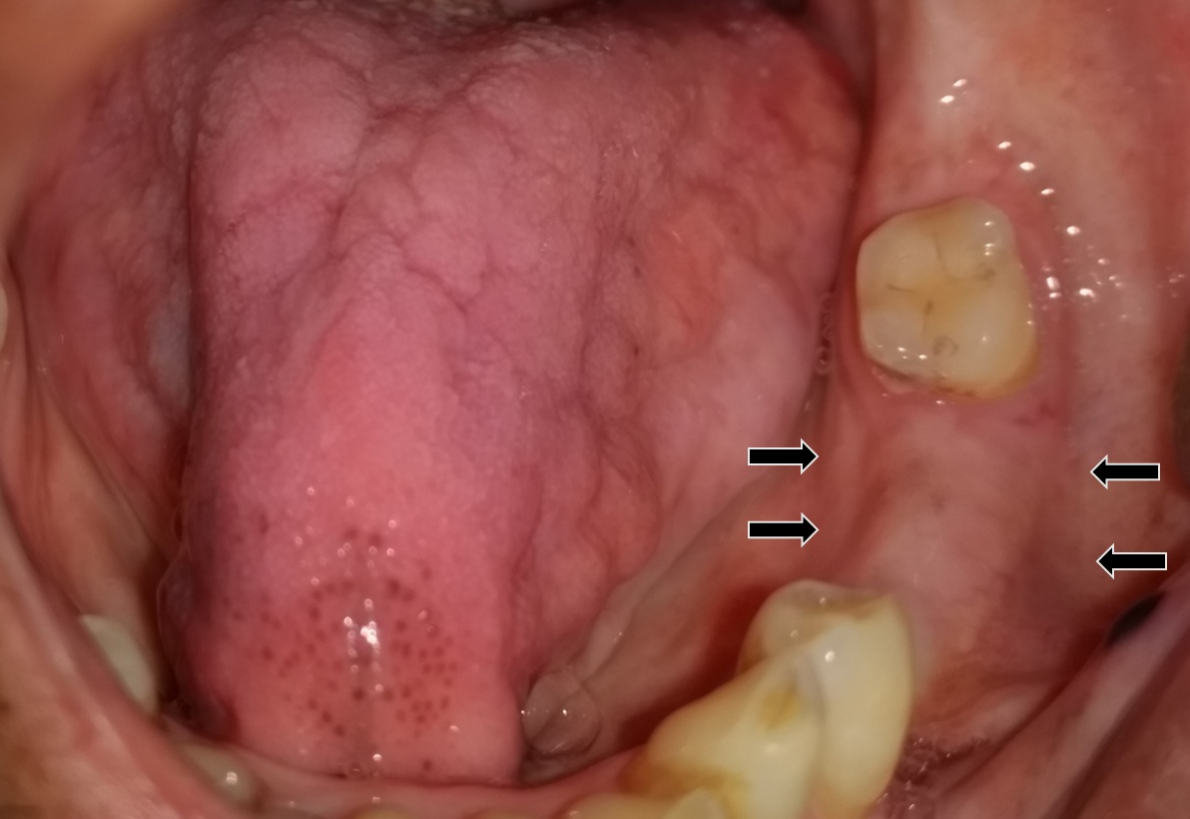
An intraoral examination revealed a mandibular alveolar swelling.

A 46-year-old man presented with a diffuse swelling in the left mandibular alveolar region since two months (Figure 1).

**Figure 2:**
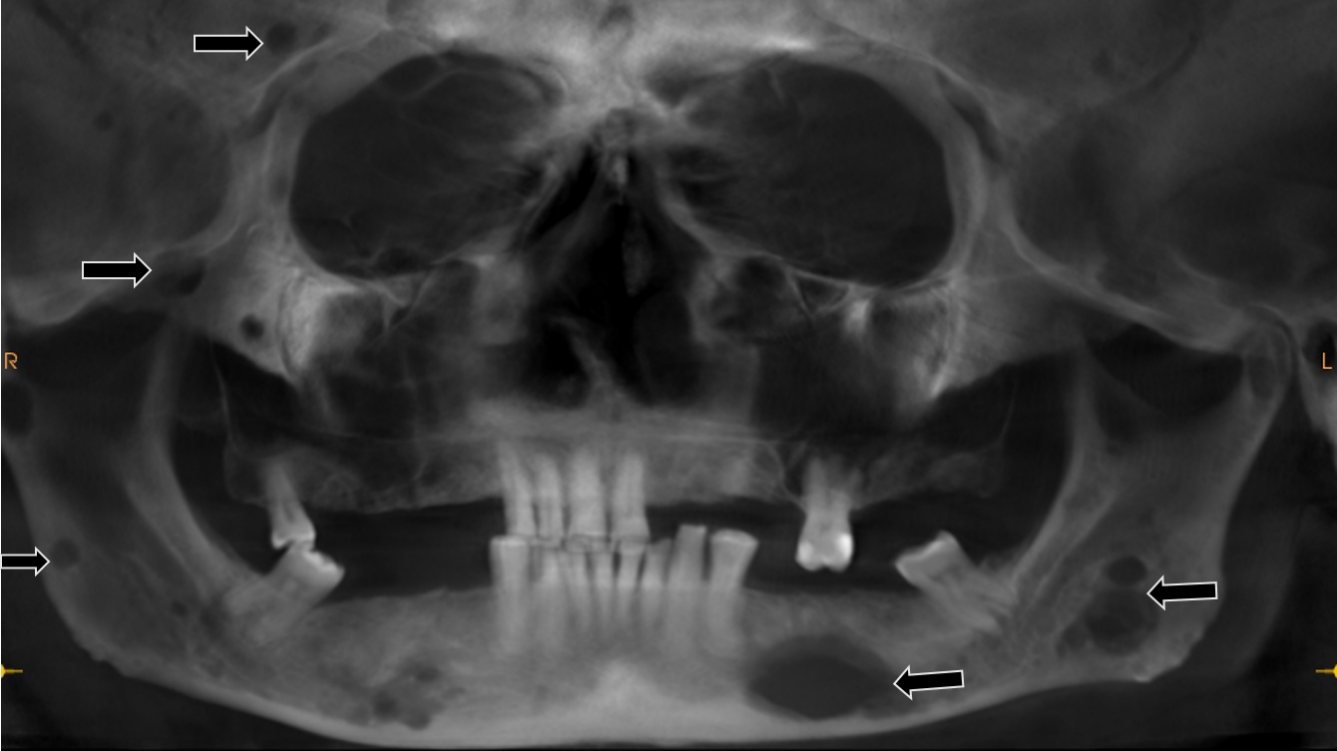
A reconstructed panoramic view showing ill-defined osteolytic radiolucent lesions in the mandible and other skull bones.

The patient revealed no history of any medical illness. On an extraoral examination, facial symmetry was noted. A swelling was noted at the medial end of the left clavicle. The left (single) submandibular lymph nodes were palpable, non-tender, and fixed. A soft, non-tender, non-pulsatile, non-hemorrhagic intraoral mass extending from the left mandibular first premolar to the mandibular second molar region was noted. A reconstructed panoramic view using cone beam computed tomography (CBCT) revealed an ill-defined osteolytic lesion in the left posterior mandible involving the inferior alveolar nerve canal and multiple punched-out radiolucent lesions indicative of multiple myeloma as a radiological diagnosis (Figure 2).

**Figure 3:**
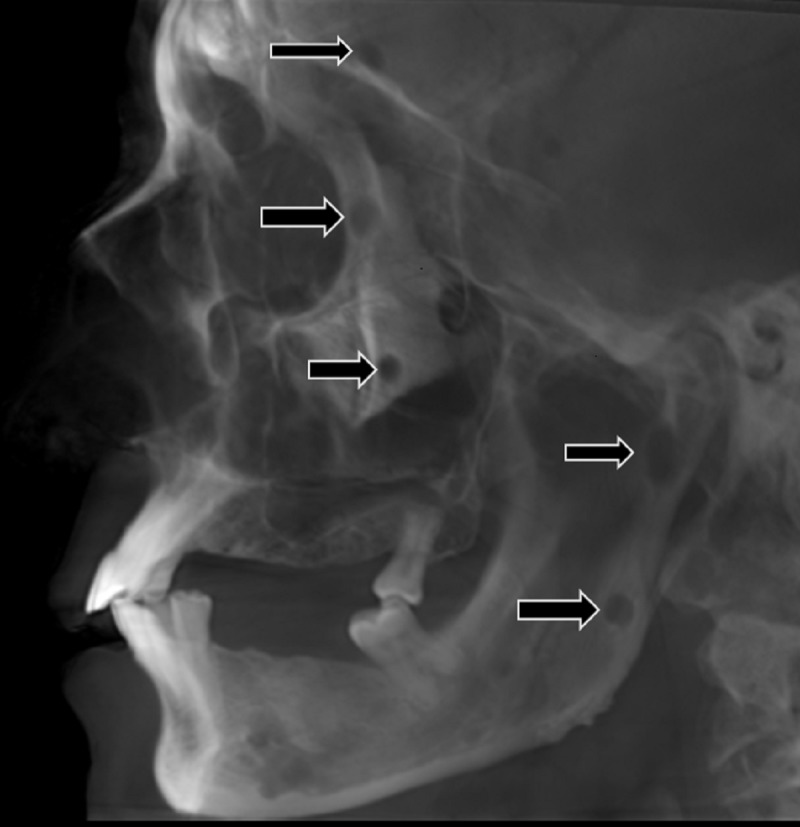
Lateral cephalogram demonstrating multiple punched-out radiolucent lesions in the mandible involving the ramus and condylar regions.

In order to establish the diagnosis of multiple myeloma, various radiographic investigations were carried out. A lateral cephalogram radiograph showed multiple punched-out radiolucent lesions (Figure 3).

**Figure 4:**
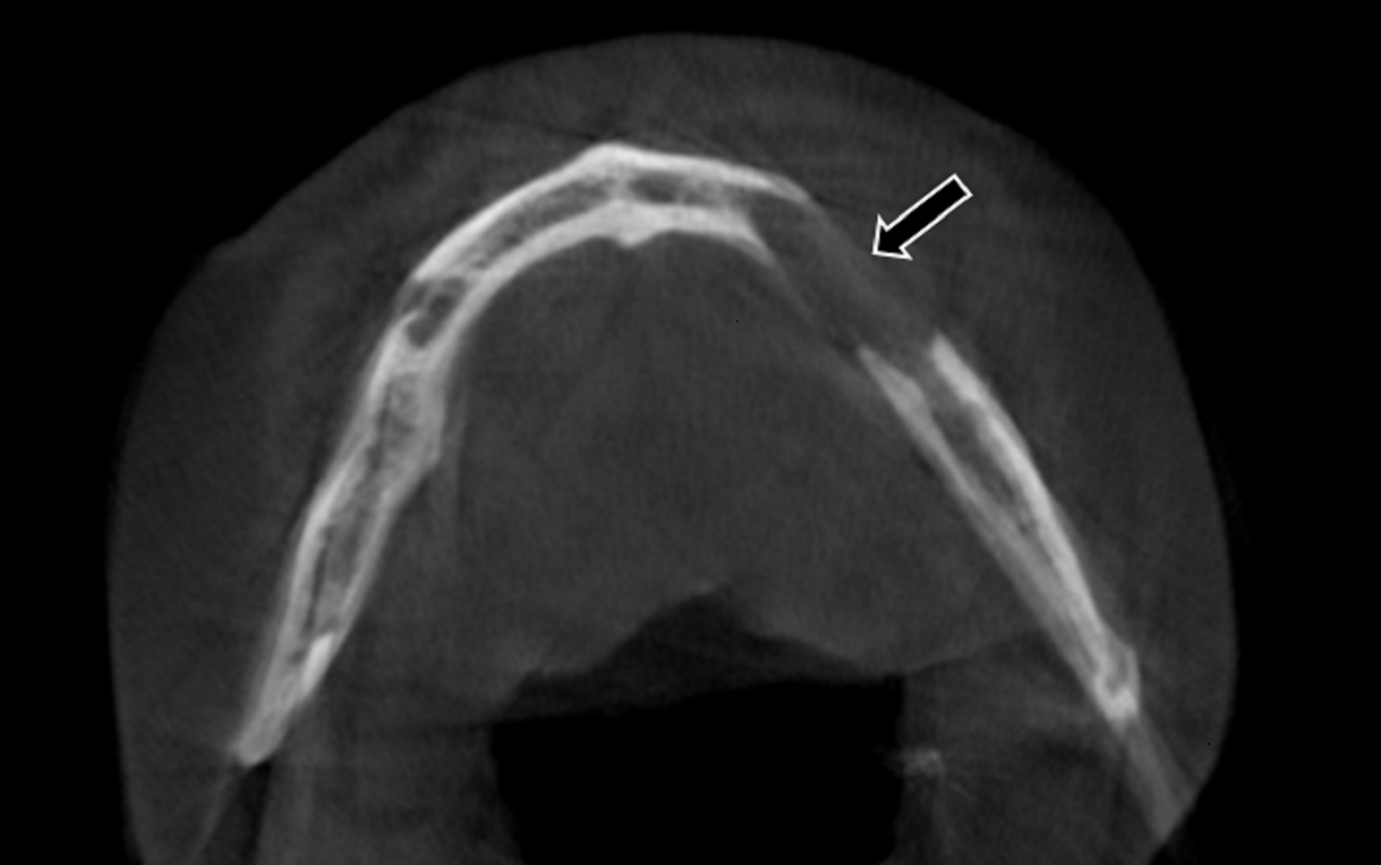
A cone beam computed tomography (CBCT) scan (axial view) showing an ill-defined radiolucent lesion in the premolar-molar region with perforation of buccal and lingual cortical plates (black arrow). CBCT - Cone Beam Computed Tomography

An axial section CBCT showed an ill-defined radiolucent lesion measuring 3.2×2.1 cm in the left mandible with loss of buccal and lingual cortex (Figure 4).

**Figure 5:**
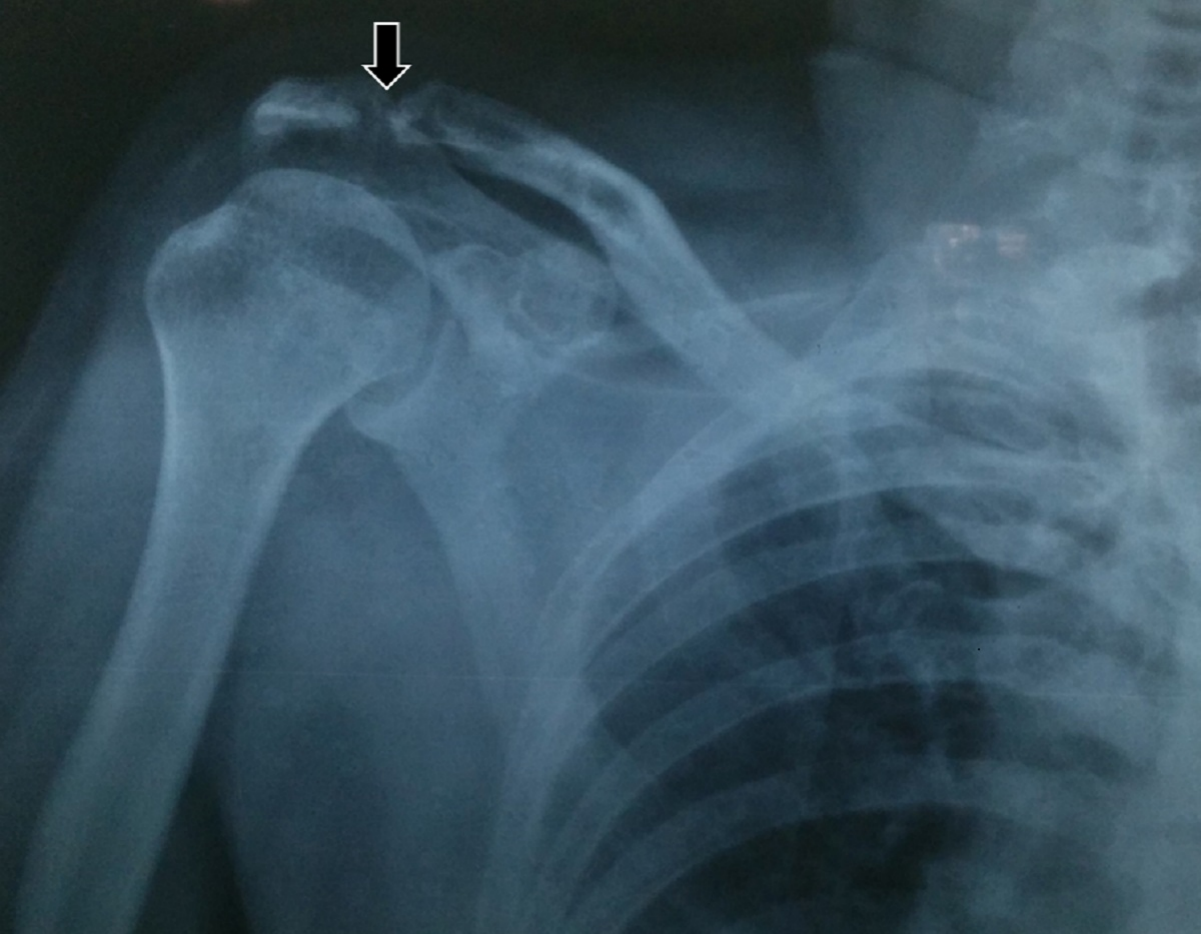
A postero-anterior shoulder view revealing a radiolucent osteolytic ill-defined lesion involving the acromioclavicular joint, suggestive of a metastatic lesion secondary to the primary lesion in the mandible.

The radiological differential diagnosis considered were multiple myeloma, browns tumor, and metastatic carcinoma. A histopathological examination of the specimen obtained from the incision showed plasmacytoma. On immunohistochemistry, the tumor cells were positive for the cluster of differentiation (CD) 138 marker and the kappa light chain. The Mib-1 (gene) labeling index was 20%-30% in the highest proliferating areas. Bone marrow aspiration showed 16% plasma cells, expressing CD38, CD138, CD56, and CD20 and was negative for CD19. Bone marrow biopsy showed trilineage hematopoiesis with an interstitial increase in plasma cells (10%). A skeletal survey showed a lytic lesion involving the left humerus, left scapula, and medial end of the left clavicle, suggestive of a metastatic lesion secondary to a primary lesion involving the jaw and skull bones (Figure 5).

The comprehensive metabolic panel (CMP) revealed serum albumin (4.3 mg/dL), serum calcium (9.1 mg/dL), and β2-microglobulin (3.87 mg/dL). The immunoelectrophoresis-serum test (IEP-S) had a faint M-band of 100 mg/dL in the gamma globulin region and immunoglobulin G (IgG) kappa (k) type on immunomodulation. Bence-Jones proteins (BJP) were present in the urine. Urine BJP was positive for κ light chains (3.19 gm/24 hrs/2500 ml urine). Cytogenetics was positive for P53 deletion in 20% of the cells. There was no evidence of t (11; 14), t (4; 14), t (14; 16), 13q deletion, or variant IgH translocation. The serum free light chains (SFLC) test showed free kappa – 19.22 mg/L and free lambda – 22.40 mg/L. Based on all the reports, including altered blood investigations, a final diagnosis of multiple myeloma and a high risk with P53 deletion was made. The patient received four cycles of bortezomib, cyclophosphamide, and dexamethasone (VCD regimen). The patient also received radiotherapy and tolerated the therapy well. Post four cycles of VCD, the urine BJP and the M band were negative. The serum free light chains (SFLC) test showed free kappa – 10.90 mg/L and free lambda – 12.60 mg/L (ratio = 1.15). Bone marrow aspiration (BMA) and biopsy were normocellular with trilineage hematopoiesis and no collection of plasma cells, suggestive of a complete response to the treatment. Cytogenetics revealed P53 deletion in 10% of the cells.

## Discussion

Multiple myeloma is the most destructive primary bone malignant neoplasia characteristically affecting the elderly, with a slight tendency to affect men. The most common early signs and symptoms of multiple myeloma are fatigue, bone pain, fever, anemia, nephropathy, and weight loss [6]. The bone marrow biopsy demonstrates a large amount of abnormal plasma cells, M-protein, light chain proteins (κ and λ), along with cytokines [7]. In our case study, bone pain was the first sign, along with swelling and the patient had no history of hemorrhage or paresthesia. The radiolucent jaw lesions occur more commonly in the mandible than in the maxilla and especially affects the molar region, ramus, angle, and condylar process, probably because of the lower amount of hemopoietic marrow in the mandible [8]. The present case had multiple punched-out radiolucent lesions in the skull. The differential diagnosis of multiple lesions with radiological findings consists of browns tumor, metastatic lesions, chronic osteomyelitis, arteriovenous malformations, and Langerhans’ cell disease. A CBCT investigation showed multiple, punched-out radiolucent lesions in the skull, which led to a radiological diagnosis of multiple myeloma. The differential diagnosis of these malignancies needs a multidisciplinary approach to diagnosis. In serum electrophoresis, M-protein is present in about 93% of the patients. In urine electrophoresis, M-protein is present in approximately 60% of the patients. The prognosis of multiple myeloma is fair with a median survival range between three and 10 years [5]. In the present case, serum electrophoresis showed an IgG monoclonal spike of 909.9 g/dL with a κ light chain, and Bence-Jones proteins were detected in the urine (3.19 gm/24 hrs/2500 ml urine).

In an advanced stage of myeloma, melphalan-prednisone plus either bortezomib or thalidomide are the novel standards in Europe for elderly patients. Thalidomide destroys malignant plasma cells directly and has antiangiogenic properties and other effects on the bone marrow microenvironment that can synergize with chemotherapy to stimulate apoptosis [9]. In the current case, the patient received four courses of bortezomib, cyclophosphamide, and dexamethasone (VCD regimen) along with radiotherapy.

## Conclusions

In conclusion, an early diagnosis of multiple myeloma of the bone is crucial to overall patient survival. This patient was diagnosed during a routine CBCT examination, and he was treated with bortezomib, cyclophosphamide, and dexamethasone (VCD regimen) along with radiotherapy. Knowledge of the maxillofacial manifestations of multiple myeloma on the part of the dental specialist is essential for an early diagnosis of the disease.

## References

[1] Pisano JJ, Coupland R, Chen SY, Miller AS (1997). Plasmacytoma of the oral cavity and jaws: a clinicopathologic study of 13 cases. Oral Surg Oral Med Oral Pathol Oral Radiol Endod.

[2] Sharma V, Sharma A (2010). Punched-out lesions in skull. Multiple myeloma. N Z Med J.

[3] Bruce KW, Royer RQ (1953). Multiple myeloma occurring in the jaws: a study of 17 cases. Oral Surg Oral Med Oral Pathol.

[4] Mozaffari E, Mupparapu M, Otis L (2002). Undiagnosed multiple myeloma causing extensive dental bleeding: report of a case and review. Oral Surg Oral Med Oral Pathol Oral Radiol Endod.

[5] Ramaiah KK, Joshi V, Thayi SR, Sathyanarayana P, Patil P, Ahmed Z (2015). Multiple myeloma presenting with a maxillary lesion as the first sign. Imaging Sci Dent.

[6] Kyle RA, Gertz MA, Witzig TE (2003). Review of 1027 patients with newly diagnosed multiple myeloma. Mayo Clin Proc.

[7] Nau KC, Lewis WD (2008). Multiple myeloma: diagnosis and treatment. Am Fam Physician.

[8] Ashcroft AJ, Davies FE, Morgan GJ (2003). Aetiology of bone disease and the role of bisphosphonates in multiple myeloma. Lancet Oncol.

[9] Hideshima T, Chauhan D, Shima Y (2000). Thalidomide and its analogs overcome drug resistance of human multiple myeloma cells to conventional therapy. Blood.

